# Adapting Agricultural Water Use to Climate Change in a Post-Soviet Context: Challenges and Opportunities in Southeast Kazakhstan

**DOI:** 10.1007/s10745-017-9947-9

**Published:** 2017-10-30

**Authors:** Tristam Barrett, Giuseppe Feola, Marina Khusnitdinova, Viktoria Krylova

**Affiliations:** 1 University of Reading, Department of Geography and Environmental Science, Reading, Whiteknights RG6 6AB UK; 2 Max Planck Institute for Social Anthropology, Halle, Germany; 3 Kazakh Institute of Geography, Almaty, Kazakhstan

**Keywords:** Adaptation to climate change, Water management, Post-Soviet transformation, Agriculture, Kazakhstan, Rapid appraisal of agricultural innovation systems, Central Asia

## Abstract

The convergence of climate change and post-Soviet socio-economic and institutional transformations has been underexplored so far, as have the consequences of such convergence on crop agriculture in Central Asia. This paper provides a place-based analysis of constraints and opportunities for adaptation to climate change, with a specific focus on water use, in two districts in southeast Kazakhstan. Data were collected by 2 multi-stakeholder participatory workshops, 21 semi-structured in-depth interviews, and secondary statistical data. The present-day agricultural system is characterised by enduring Soviet-era management structures, but without state inputs that previously sustained agricultural productivity. Low margins of profitability on many privatised farms mean that attempts to implement integrated water management have produced water users associations unable to maintain and upgrade a deteriorating irrigation infrastructure. Although actors engage in tactical adaptation measures, necessary structural adaptation of the irrigation system remains difficult without significant public or private investments. Market-based water management models have been translated ambiguously to this region, which fails to encourage efficient water use and hinders adaptation to water stress. In addition, a mutual interdependence of informal networks and formal institutions characterises both state governance and everyday life in Kazakhstan. Such interdependence simultaneously facilitates operational and tactical adaptation, but hinders structural adaptation, as informal networks exist as a parallel system that achieves substantive outcomes while perpetuating the inertia and incapacity of the state bureaucracy. This article has relevance for critical understanding of integrated water management in practice and adaptation to climate change in post-Soviet institutional settings more broadly.

**Electronic supplementary material:**

The online version of this article (10.1007/s10745-017-9947-9) contains supplementary material, which is available to authorized users.

## Introduction

Climate change is projected to have significant impacts on agriculture in Kazakhstan (Hijioka *et al*. 2014). Past trends have shown warming at rates of between 0.19 °C per 10 years and 0.46 °C per 10 years since the middle of the twentieth century, with higher temperature increases at higher altitudes (Shahgedanova *et al*. 2016). Recent climate projections from an ensemble of regional climate model (RCM) simulations suggest future mean temperature increases of up to 2 °C during 2025–2049 and of 4–5 °C in 2050–2074 (Mannig *et al*. 2013; Reyer *et al*. 2015; Shahgedanova *et al*. 2016). While there is agreement between all simulations that air temperature will increase in all seasons (although the projected magnitudes of change vary between models and scenarios), the projected changes in precipitation are spatially heterogeneous and the magnitude and even direction of change varies between seasons and models (Mannig *et al*. 2013; Shahgedanova *et al*. 2016). Analysis of the ensemble of RCM simulations, however, indicates that a decrease in precipitation during the growing season is likely (Shahgedanova *et al*. 2016). Hydrological modelling of snow and glacier melt based on future climate change scenarios suggest that freshwater limitations and water stress are among the most important likely impacts on socioeconomic and biophysical systems. National policies toward water and rural development are therefore key to responses to future climate change (Lioubimtseva and Henebry 2009; Pavlova *et al*. 2014; Pomfret 2016).

Projected impacts of climate on agriculture across Kazakhstan have been addressed by several recent studies (Fay *et al*. 2010; Lioubimtseva and Henebry 2009; Yesserkepova 2010; Mirzabaev 2013; Sommer *et al*. 2013; Bobojonov and Aw-Hassan 2014; Eisfelder *et al*. 2014). Most studies concentrate on the northern regions of the country, where large-scale industrialised grain production predominates. With low productivity and efficiency, discussion centres on whether actors are able to seize the opportunities that climate change may bring (World Bank 2009; Lioubimtseva and Henebry 2012; Bobojonov and Aw-Hassan 2014; Pomfret 2016; although Pavlova *et al*. 2014, present a less sanguine assessment). In the mountainous areas of south and southeast Kazakhstan, where agricultural production is mixed and small and medium landholdings predominate, discussion focuses on the adaptive capacity of farmers who lack financial and other resources (Lioubimtseva and Henebry 2009; Yesserkepova 2010; Kerven *et al*. 2011; Mirzabaev 2013). There is agreement that the distributional effects of climate change may likely be skewed against these areas.

Our understanding of the capacity of crop agriculture to adapt to climate change in the southern regions of Kazakhstan is limited by important gaps that result from geographical and methodological biases in the literature. First, with exceptions mostly concerning pastoralism (e.g., Kerven *et al*. 2011, 2016), these areas have been largely neglected in post-Soviet research, resulting in poor understanding of how local biophysical systems are responding to a changing climate. There is insufficient understanding of how farming is socially constructed, what institutions regulate agricultural activities, and how these may be changing concurrently or responding to climate change. Second, research tends to be based on top-down climate, crop-climate, or climate-economic modelling, engaging little with communities on the ground and making limited use of social scientific theories to examine adaptation practices, or lack thereof, in specific places (Lioubimtseva and Henebry 2009; Hamidov *et al*. 2016). Research has focussed on technological or managerial adaptation measures (drought resistant crop varieties, insurance schemes) (e.g., Heidelbach 2007; Pavlova *et al*. 2014) at the expense of cultural or social adaptation practices, which are not only more difficult to model, but also call for critical analysis of the socio-political context, usually a given external factor in climate and economic models (Feola *et al*. 2012, 2015).

This study provides a place-based analysis of constraints and opportunities for agricultural adaptation to climate change, with a specific focus on water use in two districts in southeast Kazakhstan. We focus specifically on crop farming; the effects of climate change on Kazakhstan’s extensive livestock sector are beyond the scope of this research. The convergence of post-Soviet political, economic, and socio-cultural environments and climate change has been underexplored so far, as have the consequences of such convergence on agriculture in Kazakhstan and Central Asia. This paper is the first account of on-the-ground adaptation to climate change in Kazakhstan and the first to consider how the post-Soviet institutional environment converges with and affects adaptation to climate change, and to what extent implemented water management models (particularly IWRM) support climate change adaptation in a semi-arid region. These findings have broad relevance for other former Soviet countries and countries that have undergone major institutional transformation from long-established regimes worldwide.

## Agriculture and Water Management in Post-Soviet Kazakhstan

Agriculture is an important sector of Kazakhstan’s rural economy, accounting for 80% of land-use, 4.4% of GDP, and providing livelihoods for some 19% of the labour force (World Bank 2016). The dissolution of the Soviet system led to fundamental and ongoing restructuring of political and economic institutions, connections to regional and global markets, and social relations, and in the agricultural sector to widespread de-intensification as without Soviet-era inputs and subsidies, pastures and marginal arable land were abandoned (de Beurs and Henebry 2004). State support for agriculture was terminated and many farms became heavily indebted amid a tortuous land reform program aimed at restructuring and privatising former state and collective farms (Pomfret 2013).

Land reforms during the 1990s had little to no impact on the size and internal structure of large farms. But a bankruptcy law enacted in 1998 led to the liquidation of insolvent corporate farms and an increase of private farms from approximately 5000 in 1990 to 162,000 in 2006 (Wegerich 2008; World Bank 2004). The adoption of a new land code in 2003 introduced private ownership of farmland and a fledgling land sales market (Petrick *et al*. 2011). Kazakh law now recognises three farm structures: household producers, individual farms, and corporate farms. Large agro-holdings and agro-food clusters enjoy unclear legal status (Wandel 2009). Household producers and individual farms account for the majority of agricultural production (39% of arable land and 71% of gross agricultural output in 2010) (Lerman 2009, 2014).

Post-Soviet farmers generally have a negative attitude to the removal of agricultural subsidies and the institution of land markets. In several former Soviet countries, farmers and rural populations did not support cropland privatisation and opposed private land ownership and markets (Lioubimtseva 2010), attributing responsibility for food security to the state and consequently blaming it for poor economic conditions and increasing food prices (Serova 2000). Various issues exacerbated dissatisfaction, including high transaction costs (bribes, administrative obstructions), restricted access to finance for agribusinesses, low land productivity, land tenure restrictions, and an underdeveloped transport infrastructure in grain production (Meng *et al*. 2000; Petrick *et al*. 2014: 1, citing OECD 2011, 2013). Moreover, as DFID (2003: 72) noted, a consequence of privatisation and redistribution of land to former state and collective farm employees is that “many current farmers have relatively little experience in general farm management practices, either because they are new to farming or because their jobs on the state and collective farms were specialised.”

### Integrated Water Resource Management and Irrigation Management Transfer

Soviet-era water management for agriculture and energy was centralised under the all-Union Ministry of Melioration and Water Management (*MinVodKhoz*), its corresponding ministries and subordinated agencies in the republics. The concentration of agricultural production in arid and semi-arid areas – with otherwise favourable soil and thermal conditions – necessitated extensive water storage and distribution systems oriented to planned production by state enterprises that had responsibility for on-farm water delivery. These systems were of low technology and efficiency (50–60% in Kazakhstan) and by the 1980s in severe need of rehabilitation (Micklin 1988: 17).

One of the most significant consequences of the dissolution of the Soviet system has been the opening of the country to international development assistance, with donors promoting the transfer of privatised models of natural resource management. Among these, Irrigation Management Transfer (IMT) “seeks the relocation of responsibilities and authority from central governments entities managing irrigation schemes to non-governmental agencies such as the Water Users Associations (WUAs) or to private entities” (Zinzani 2015:767). IMT is the backbone of Integrated Water Resource Management (IWRM), a hegemonic paradigm elaborated in the 1992 Dublin principles to promote sustainable social and environmental development through water management, formalised in Kazakhstan in the 2003 Water Code.

Evidence suggests that the implementation of IMT and IWRM in Kazakhstan has not been informed by a solid understanding of the local cultural, socio-political, and economic context (DFID 2003; Wegerich 2008; Mukhtarov 2013; Zinzani 2015). Some authors have noted the inflexible implementation of IMT and other resource management models (Wegerich 2008), and lack of consideration in development orthodoxies for place-specific conditions (Kerven *et al*. 2011, 2016; see also Meinzen-Dick 2007). This is consistent with evidence in other countries in the global South (Biswas 2004; Solokov 2006; Meinzen-Dick 2007; Mollinga *et al*. 2007), and with typical dynamics of policy implementation in Kazakhstan (Petrick *et al*. 2014) where the “reform process has been characterised by variability and arbitrariness at the local level” (Behnke 2003), which is similar to implementation of IWRM in neighbouring former Soviet countries (Hornidge *et al*. 2011).

Water Users Associations (WUAs) were established after 1994 with the intention of creating agricultural water markets (Burger 1998), although this often entailed merely a change of name of pre-existing structures and infiltration of decision structures by local farm elites, with attendant issues over accountability, lack of specialist knowledge, and representation of farmer interests (Wegerich 2008; see also Zinzani 2015).^1^

A second important issue was the inability of WUAs to convert an irrigation system designed for a large-scale farming system and which collapsed with the fragmentation of farms. Failure to maintain or upgrade water storage and irrigation infrastructure resulted in reduced capacity of canals, especially smaller canals, and in high water losses (Wegerich 2008; Hornidge *et al*. 2011). WUAs often lack the financial resources required for infrastructure investment, as not all farmers regularly pay their fees and transfers from central and district government have been reduced or stopped (Burger 1998; Johnson 1998; Rosen and Strickland 1998; Zinzani 2015).

Persistent top-down decision-making and broader unequal relations also inhibit efficient water use in agriculture. In Kazakhstan, district water departments often override WUAs decisions about maintenance work, while unequal relations (e.g., between small and large landholders) influence not only how conflicts regarding water are addressed, but whether they are raised in the first place. Rigidities in the water management system therefore result from both infrastructural path dependence and persistent power relations and other social dynamics resulting from the fragmentation of decision-making actors following the collapse of the Soviet agricultural sector (Van Assche *et al*. 2014).^2^

## Methodology

This study set out to investigate factors affecting agricultural adaptation to climate change in southeast Kazakhstan with four specific objectives: (i) characterise the water systems in representative sites; (ii) identify the challenges faced by a range of actors involved in water use in agriculture; (iii) identify current water use and management practices employed to deal with water stress and variability; (iv) identify entry points for adaptation of water use in agriculture. Here we briefly present the approach used to achieve these objectives (for a fuller presentation and critical discussion see Barrett *et al*. 2017).

### Data

Fieldwork was conducted in three phases between September 2015 and March 2016. Data were collected using an adapted Rapid Appraisal of Agricultural Innovation Systems (RAAIS) approach (Schut 2014; Schut *et al*. 2015a, b, c, d, 2016). RAAIS “is a diagnostic tool that can guide the analysis of complex agricultural problems and innovation capacity of the agricultural system in which the complex agricultural problem is embedded” (Schut *et al*. 2015a:1). Emerging from agricultural innovation studies within a broader Farming Systems Research approach (Darnhofer *et al*. 2012; Klerkx *et al*. 2012) RAAIS is a multidimensional, multilevel, multi-stakeholder, and participatory tool for assessing the innovation capacity of an agricultural system. It combines “qualitative and quantitative methods, and insider (stakeholders) and outsider (researchers) analyses which allow for critical triangulation and validation of the gathered data” (Schut *et al*. 2015a:1). Accordingly, we employed the following complementary data collection methods:

#### Multi-stakeholder Participatory Workshops

We conducted one-day workshops in each of the two study sites in February 2016. These brought together approximately 30 people each in separate stakeholder groups of small farmers, medium and large-scale farmers, non-governmental organisations (NGOs), scientists, agribusinesses, and representatives of state structures (e.g., local and regional authorities, WUAs) to discuss the theme: *Water-use in agriculture: main challenges and ways forward*. The workshops consisted of seven exercises:

1. Individual brainstorming of challenges and constraints
2. Ranking constraints to adaptation within stakeholder groups
3. Identifying nature of constraints (technological development, government programmes and insurance, farm production practices, farm financial management)
4. Categorising type of constraints (understanding, planning, management)
5. Situating constraints and challenges at different levels (farm, village, district, region, national, international)
6. Identifying linkages between constraints and identifying key constraints
7. From constraints and challenges to entry points and best bets for adaptation

Exercises 2 to 5 were conducted within the stakeholder groups, while groups were brought together for Exercises 6 and 7. We adapted the methodology to suit our theoretical framework and objectives. The typology of adaptation used in Exercise 3 followed that proposed by Smit and Skinner (2002), which defines different types of agricultural adaptation measures, and was used to identify types of constraint faced by different actors; participants were asked whether those constraints were problems of (i) technological development, (ii) government programmes and insurance, (iii) farm production practices, or (iv) farm financial management (Barrett *et al*. 2017). The understanding-planning-managing framework for Exercise 4 was derived from Moser and Ekstrom’s adaptation process framework (2010), which conceptualises a generic process of deliberate adaptation of the type expected in agricultural water use. Thus, participants were asked whether the five constraints and challenges collectively selected and ranked in Exercise 2 represented problems of (i) understanding (i.e., not enough known about what is going on in the water system to be able to change/adapt), (ii) planning (although knowing what is going on, have difficulty deciding and planning what to do to change and improve water use), or (iii) managing (knowing what is going on and what they want to do, but not knowing how to implement water-use changes and adaptations) (Barrett *et al*. 2017).

#### Semi-structured in-Depth Interviews

Follow-up interviews were conducted with workshop participants following an opportunity sample design whereby farmers were purposefully over-represented (10 out of 21 interviewees) to gain better insight of the experiences and perspectives of water users. Interviews were conducted on a one-to-one basis, and focused on the structure (physical and institutional) and functioning of the water system, and on current adaptation practices from the interviewee’s perspective. Thus, interviews complemented the workshops. Interviews were guided by the research team on the basis of a flexible protocol in order to insure, on the one hand, that the interview maintained the above mentioned focus, and on the other, that the interviewees had sufficient freedom to propose their own perspectives or interpretations, and raise any connected points for understanding the water system and current adaptation practices. Each interview lasted approximately one hour and was digitally recorded. Interview notes were compiled with inputs from the two or three interviewers who conducted each interview.

#### Secondary Data Collection

Secondary data were collected from the National Agricultural Census of 2006/2007, district and regional authorities (background interviews, annual reports, statistical databases), and agricultural statistics from the Kazakh Ministry of National Economy. These data mostly consist of standard agricultural indicators aggregated at the regional level (e.g., agricultural land, number of farm enterprises, irrigated land), and are therefore useful only as a broader contextualisation of data from the multi-stakeholder workshops and semi-structured interviews.

### Study Sites

**Fig. 1:**
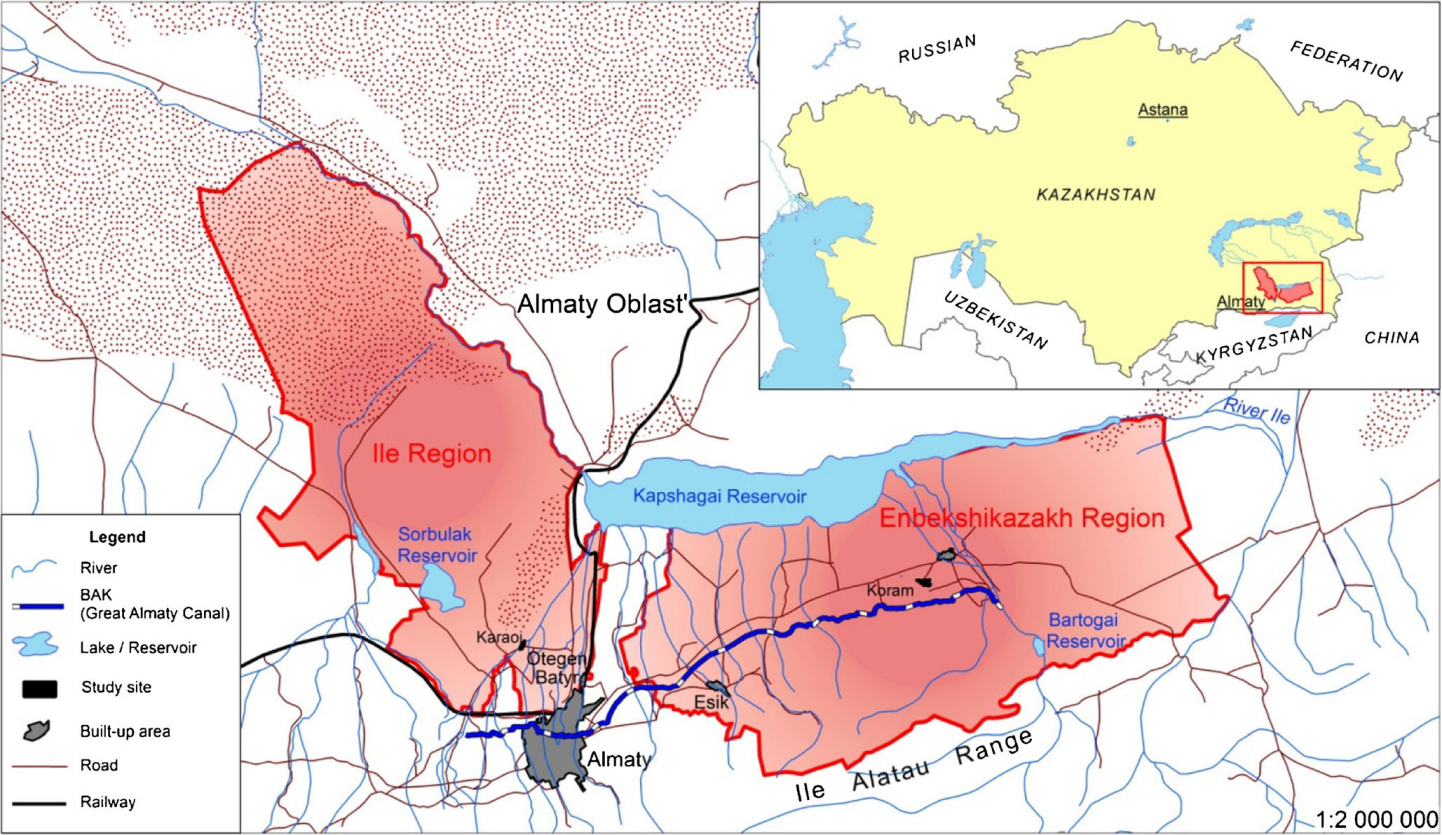
Map of the study area

We selected two study sites in different regions of Almaty Oblast’, southeast Kazakhstan, to represent broadly ‘typical’ water management sites fed by the Ile Alatau mountain system, with a mix of irrigated and rain-fed agriculture. The climate is extremely dry, harsh continental, with average temperatures ranging from −7 °C in January to 22–25 °C in July and an average of 150–170 frost-free days per annum. Rainfall varies with elevation across the region from 200 mm on the plains to 970 mm at the high-elevation Tuyuksu station located at 3440 m. Locations were selected Key differences between sites were geographical location, farm size, and proximity to the main market in Almaty (Fig. 1).

Koram (population 6000) is a former tobacco producing state farm [*sovkhoz*] within Enbekshikazakh Region. It lies on the plain below the northern foothills of the Ile Alatau, 10 km southwest of the former regional centre, Shelek, and 130 km east of Almaty. Koram comprises some 7700 ha of land, now privatised into individual farms. Most households are engaged in farming of some sort: Koram has 833 registered farms and 1005 agricultural households (MA n.d.).

Agricultural land accounts for some 4682 ha, of which 58% (2723 ha) is irrigated arable land and 37% (1753 ha) is pastures. The rest is given over to haymaking (203 ha) and a small amount of multi-annual crop production (4 ha) (KAGIS 2016). Farms tend to be small (less than 25 ha), mixed arable and livestock, with most arable production (maize, alfalfa, clover) used as fodder for cattle, horse, sheep and poultry production. In addition, small-scale vegetable production (peppers, tomatoes, cucumbers) is done on privatised land-shares. The village also lies sufficiently close to the mountain system to benefit from precipitation favourable for orchard production. Produce is sold wholesale to Russian shuttle traders, via local middlemen to the wholesale market in Almaty, or by limited roadside sales.

Karaoi (population 5600) is the administrative centre of Karaoi rural district in Ile Region, some 20 km northwest of Almaty and 25 km from the regional administrative centre, Otagen Batyr. The region’s landscape is largely represented by semi-deserts, used for dryland farming on the Karaoi plateau and winter, spring, and autumn pastures in the Sartaukum desert. Karaoi is one of the largest districts in Ile Region, comprising three villages and a territory of 8570 sq. km (OEBPIR 2015).^3^ Farms range from household plots of 0.5 ha to greater than 3000 ha and comprise a range of organisational forms including a single production cooperative, six business partnerships, 197 individual private farms, and 1343 farming households (OEBPIR 2015: 25; MA n.d.). There is also significant formal and informal rental of land plots and unregistered small-scale farming activity.

Agricultural land in Karaoi accounts for some 53,737 ha, of which 26% (14,090 ha) is arable and 74% (39,647 ha) is used for pasture and haymaking. Around 14% (2035 ha) of arable land is irrigated (KAGIS 2016) and production consists mostly of maize for human and animal consumption, soy, wheat and alfalfa, as well as oil, potato, and vegetable production. Winter wheat, winter and spring barley, safflower, and wheatgrass are grown on rain-fed arable land, mostly as fodder. Closer to the city, agricultural production is more vertically integrated than in Koram. The region boasts four processing plants for animal products and crops. Nevertheless, production is primarily for local markets. Even small-scale production is often oriented to local processing plants (flour, sugar, poultry). Smaller producers sell to established networks of clients, at a seasonal market in Karaoi village, and at the wholesale market in Almaty.

## Results and Analysis

### Irrigation Systems in Koram and Karaoi

**Fig. 2:**
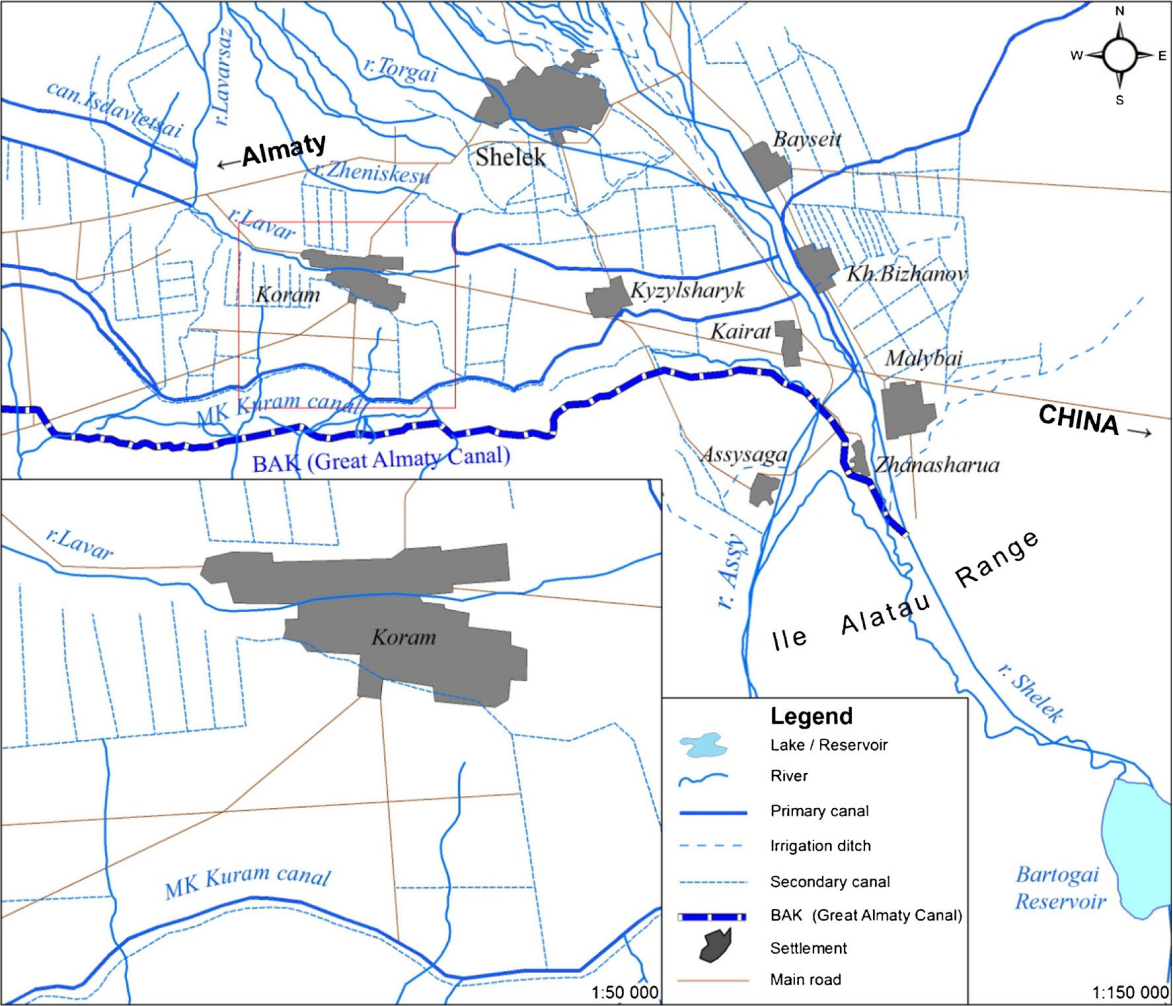
Map of water system in Koram

**Fig. 3:**
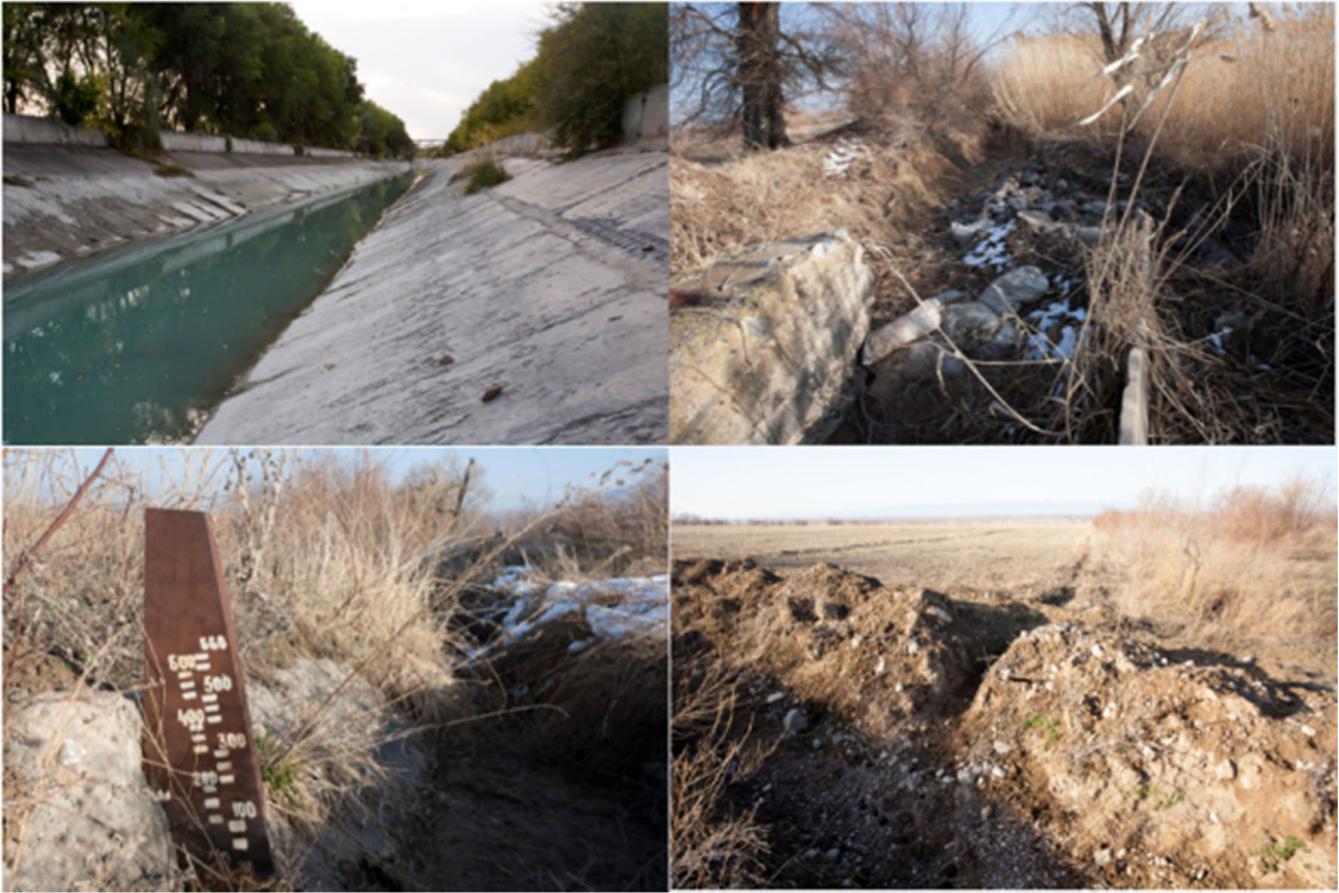
Water system in Koram. Clockwise from top-left: (1) BAK, (2) MK Kuram, (3) water meter, (4) on-farm canal and irrigation ditch

The primary water source for Koram’s irrigation system is the Great Almaty Canal [*Bol’shoi Almatinskii Kanal*] (BAK) (Figs. 2 and 3) a large capacity concrete canal that was built in the late-Soviet period to increase irrigated agriculture in the area. The canal is fed from the Bartogai reservoir and is managed by a filial of a state enterprise tasked with agricultural water management under the Ministry of Agriculture, KazVodKhoz. Water from the BAK is distributed via feeder canal to a large earthen primary canal, the MK [*magistral’nyi kanal*] Kuram, under the administration of the local WUA. Dug by hand in the 1940s, the MK Kuram has suffered from decades of non-maintenance (Fig. 3). A series of sluices connects this to an earthen on-farm channel [“*vnutrokhoznyi*” *kanal* 4], in effect little more than a ditch, and associated network of ditches [*aryki*] by which channels water onto the fields. Primitive water gauges are installed at the sluices connecting the MK Kuram to the on-farm canal (Fig. 3), and water is distributed to particular fields by opening and blocking ditches with sandbags and boulders. On-farm water distribution is the responsibility of farmers, although it is in practice negotiated in conjunction with the head of the WUA. Disagreements have occasionally arisen and individual farmers have been known to take matters into their own hands to obtain their share of water. A second water supply comes from the Terekti, a mountain stream that irrigates a patch of pastureland west of the village centre and is channeled along a similar system of ditches. Farmers who rely on this water source report no problems with irrigation.

Koram’s WUA was established in 2003 by the present head of the village administration and has its offices in the village’s administration building [*akimat*]. It acts as an intermediary between 740 local farmers and the BAK. Farmers close an agreement with the WUA on a yearly basis in March and agree a price according to the type of crop and area under cultivation. The WUA then contracts with the BAK on behalf of farmers and agrees a delivery date. Farmers pay half the money up front and half at the end of the growing season. The WUA also coordinates farmers’ annual upkeep of the district irrigation network (MK Kuram, secondary canal, and ditches) and contracts for heavy maintenance work. The director and his six staff are funded 80% by farmer’s water payments and 20% by state subvention.

**Fig. 4:**
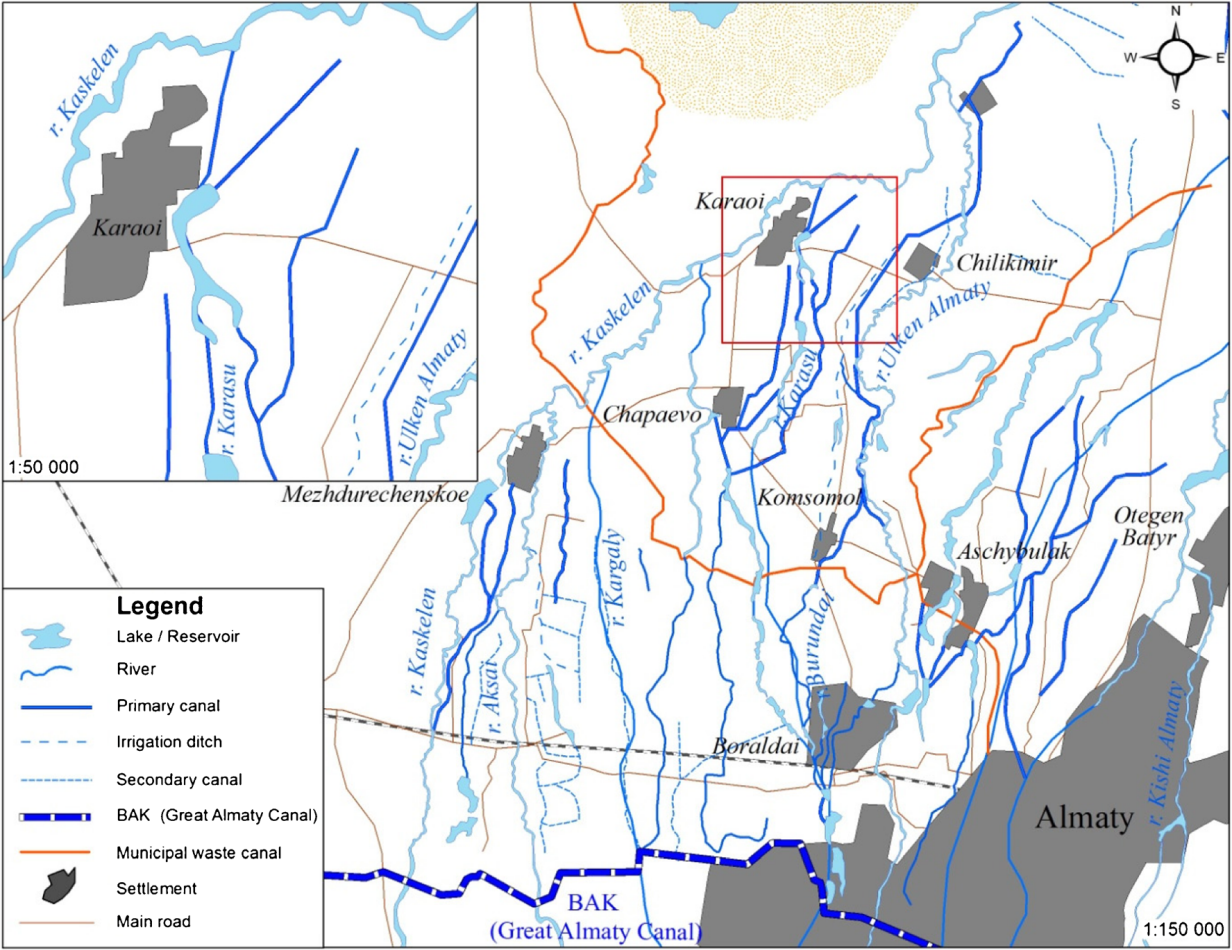
Water system in Karaoi

**Fig. 5:**
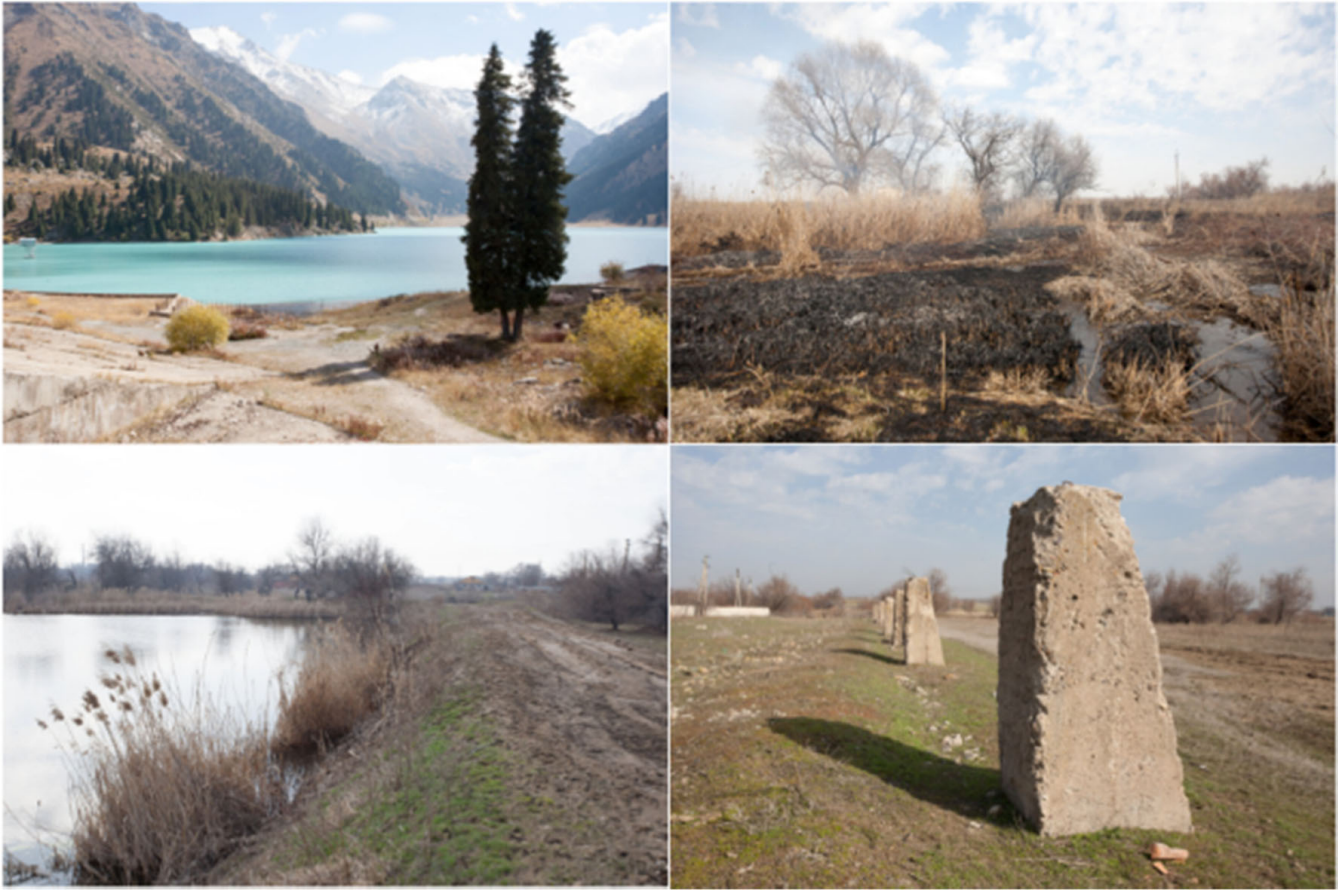
Water system in Karaoi. Clockwise from top-left: (1) Glaciers in the Ile Alatau mountains, the source of the Ulken Almatinka, (2) burning reeds to clear irrigation channels, (3) water collector pond, (4) dismantled Soviet concrete irrigation canal

In Karaoi, water for agricultural production is obtained from several sources (Figs. 4 and 5). The Kaskalen and the Ulken Almatinka rivers account for four fifths of water supply. The BAK represents another source. A municipal wastewater treatment plant in Almaty provides water for the production of feed crops and is distributed along a separate 18 km canal. Groundwater is also obtained from (mostly Soviet-era) wells and used for livestock production on non-irrigated farms.

Water from mountain rivers and the BAK is channelled through a system of earthen primary canals administered by the semi-private production cooperative [*proizvodstvenii kooperativ*] PK “Ili,” and collected into a network of collector ponds (Fig. 5). Water is distributed from these ponds via sluices into a network of earthen on-farm channels and thence to ditches to particular fields (Fig. 5). As in Koram, these on-farm channels and ditches are the responsibility of local farmers.

Karaoi’s irrigation system differs from Koram in terms of the number of actors involved. The local WUA (“Kos-Ozen”) covers five villages and is considered to be a branch of Ili Irrigatsiya, a municipal utility service [*gorodskoe kommunal’noe prepriyatie*] based in the district centre, Otagen Batyr. Karaoi’s irrigation system is larger and more vertically integrated than Koram’s. The local WUA consists of one employee with responsibility for five villages who is accountable to Ili Irrigatsiya.

Functionally, these are extensions of the district administration. In conjunction with the WUAs, Ili Irrigatsiya agrees contracts with farmers, acts as an intermediary for supply requests from the BAK, and administers an annual irrigation schedule. This irrigation schedule covers provision from primary canals and collectors. On-farm irrigation is negotiated between farmers. As in Koram, farmers agree delivery contracts with Ili Irrigatsiya, and pay 50% of water charges at the start of the irrigation season and 50% after harvest.

### General Patterns in Identification of Challenges

Key challenges identified during the workshop in Koram related to one of three categories: (i) water supply (timing, inadequacy, and unclear supply rules), (ii) poor infrastructure (condition and maintenance, lack of consumption measurement equipment), and (iii) high water prices. Problems falling into these categories were mentioned most frequently in Exercises 1 and 2 and were ranked highest by workshop participants above other constraints, such as climate change, soil cultivation practices, knowledge transfer, or water losses (Appendix).

Some challenges highlighted in Koram also featured prominently in Karaoi, where participants were overwhelmingly concerned by: (i) water supply (inadequacy), (ii) poor infrastructure (condition and maintenance, but not consumption measurement equipment), and (iii) lack of irrigation technology (drip and sprinkler irrigation) (Appendix). This last challenge, which was not mentioned in Koram, can be explained by the presence of larger and more mechanised farms in Karaoi. Water supply rules were mentioned frequently by individual participants, but were accorded less prominence when prioritising challenges in stakeholder groups.

Climate change was not perceived to be significant in either study site: it was mentioned only a few times by actors not directly involved in local agriculture (NGOs and scientists). However, there was widespread perception of declining water inflows in both sites. The water stock of the Bartogai reservoir was reportedly depleted in 2012 and 2014, affecting farmers reliant on the BAK. In Karaoi, decreasing water stock was reported from the Ulken Almatinka and other mountain rivers. Farmers and WUA officials report that water flow through this river system has declined, small channels have dried up, and some collector ponds have become so silted as to be almost unusable. These reported decreases contradict discharge measures in the undisturbed parts of the mountain river system, indicating that perceptions of decreasing supply occur in a context of increasing demand. Analysis of long-term observations of the headwaters of the Ulken Almatinka since 1952 shows increased discharge in all seasons including summer as glaciated catchments melt (Shahgedanova *et al*. 2016). The BAK is asked to provide for an increasing area of land as agriculture continues to recover from the collapse of the 1990s. The section of the BAK that includes Koram irrigates 9300 ha of land; up from 3500 ha in the 1990s but still less than the 17,000 ha in the Soviet period. The Ulken Almatinka used to irrigate 3000 ha in Karaoi, but now feeds only 1200 ha.

Farmers also mentioned climate change with respect to snow cover. In rain-fed zones a good year depends on snow lying on the ground during the previous winter. This has become increasingly problematic. The first snow in the winter of 2015–2016 was reported to be very late, and winter air temperatures were as high as 15 °C, so little snow remained on the ground.

Participants in Koram (smallholders, state officials) highlighted uncertainty and lack of clarity around existing water supply rules, while participants in Karaoi mostly discussed noncompliance. Farmers and NGOs mentioned non-compliance in interviews, but not state officials (water users associations, local administration, BAK). Transgressions were reported in abstraction of water without payment or outside of mutually agreed supply rotations, diversion of water from other farms, and higher-level diversion of state funds earmarked for investment in water supply infrastructure.

Finally, in both sites participants took the Soviet period as a usually positive benchmark by which to assess the current water system. Local actors considered adaptation in terms of restoring the Soviet irrigation system. For example, a BAK representative clarified the local administration’s aim as being “to repair [unmanaged canals], reduce water losses, restore irrigated lands, and rebuild the system as it was in the Soviet Union.” Similar attitudes were evident when actors compared the area of irrigated land with Soviet-era benchmarks, taken as a target for re-irrigation. This baseline refers to a period when “there was still a system” and when agricultural productivity was very clearly higher. This, of course, depended on a whole socio-technical system of subsidised inputs that allowed for higher productive capacity.

### Differences Among Actors in Identification of Challenges

In both Koram and Karaoi small and large landholders concurred on some challenges but diverged on others, which revealed their distinct experiences. Further, in both study sites there was a substantial overlap of challenges identified by farmers and state structures. In Koram, small and large landholders and state structures substantially agreed on the three challenges listed above, but placed different emphasis on *unclear supply rules* (smallholders and state structures), *untimely supply and high prices* (small and large landholders), *poor infrastructure and maintenance* (large landholders and state structures), and *lack of consumption measurement* (state structures) (Appendix). Similarly, in Karaoi small and large landholders concurred in stressing *inadequate supply* and *poor infrastructure and maintenance*, but differed in emphasising *supply rules* (smallholders), and *lack of technology* (large landholders), while state structures also strongly highlighted *poor infrastructure and maintenance* (Appendix).

In both study sites non-local actors did not experience water use directly, identified challenges that differed significantly from those identified by local actors (small and large landholders and state structures). In Koram, for instance, NGOs, agribusinesses and scientists identified a wider range of challenges, including supply rules, water losses and inadequate supply, lack of knowledge, and land use. In both Koram and Karaoi NGOs, agribusinesses and scientists mentioned a more diverse range of challenges than farmers and state structures, which mentioned fewer problem areas, showing a stronger in-group consensus (Appendix). These differences may have been determined by the relative distance of these actors from direct and daily experience of water management, but also by a distinct conceptualisation and understanding of water use shared by both scientists and NGO representatives.

### Categorisation of Challenges

All participants in the two workshops in Koram and Karaoi classified the majority of their challenges (approximately 60%) as related to *government programmes* rather than technological development, farm production processes, or farm financial management. Similarly, all actors considered that of the challenges identified most (48% in Koram, 60% in Karaoi) related to *management* of water stress, while only a few were related to understanding the nature of experienced water stress or planning for better water management.

Another similarity is that actors tended not to situate challenges at their own system level, but at levels below or above them. In Koram, 60% of challenges were situated by participants at local level (farm or district). NGOs and agribusinesses, scientists, and state structures located issues at farm level, but farmers did not. State structures located issues at national level (i.e., government policy), while farmers located them at the level of village and district (smallholders) or district and region (large landholders). NGOs, agribusiness, and scientists located issues at various levels. Also, farmers (both small and large landholders) and state structures situated challenges at fewer levels (i.e., showed more in-group consensus) than other actors. In Karaoi 64% of challenges were situated at village to regional level and no challenges were situated at farm level by any actor, while the key challenges identified in Exercise 6 were situated at regional level (Almaty Oblast’).

#### Spatio-temporal Distribution of Challenges and Problems

Participants in both Koram and Karaoi emphasised that water stress is unevenly experienced during the year, as it is especially felt in the summer at the height of the growing season. Most importantly, it was apparent that the challenges faced by farmers strongly depended on the location of their plots. The presence of reservoirs or groundwater in Karaoi offered alternative sources of water for irrigation that could compensate for lower inflows from managed and unmanaged secondary canals or lack of connection to those canals provided the farm had sufficient financial capital to purchase and operate pumps to extract water. In Koram, farmers who did not depend on water supply via publicly managed and unmanaged canals were less concerned with water stress. Further it was apparent that the challenges faced and the level of concern with water stress is markedly different among farmers whose plots were located upstream (i.e.,closer to the head of the MK Kuram and secondary on-farm canal) and those with downstream plots as the latter often received less water or received water with more substantial delays.

### Current Water Management and Adaptation Practices

Most management and adaptation practices were implemented by farmers and WUAs, that is, by the actors most directly involved in water use in agriculture (Table 1). Farmers appeared to be engaged mostly in day-to-day operational practices, such as offsetting water scarcity through alternative water sources. WUAs appeared to facilitate collective action among farmers by coordinating water supply rotation in periods of drought and the voluntary maintenance and cleaning of ‘unmanaged’ canals, usually in spring before the start of the irrigation season (end of April). In 2014, for instance, when Koram received only two fifths of the requested water supply, the WUA reduced the area of land to be irrigated and channelled water from three out of seven sluices, working day and night to distribute evenly and in time to prevent crop failure. The strategy was to distribute water evenly, but to reduce the volume given to individual farmers to ensure that everybody got a crop, if not an optimal yield. According to the head of the WUA, “the farmers weren’t happy, but they understood.” The voluntary maintenance and cleaning of ‘unmanaged’ canals had a tactical (i.e. seasonal) rather than structural value; in the absence of adequate capital for a refurbishment and upgrading of the canals, the seasonal intervention was limited to basic maintenance aimed at facilitating water flow and reducing water losses.

**Table 1:** Adaptation practices implemented in Koram and Karaoi

Practice	Purpose	Type	Timeframe of effects^a^	Actor	Location where practice reported
Use of alternative water supply (e.g. underground water)	To make up for lower inflow from canals	Technical	Operational	Farmer	Koram, Karaoi
Additional use of fertilisers	To make up for lower inflow from canals	Technical	Operational	Farmer	Karaoi
Illegal access to water (e.g. diversion of water from other farmers‘canals)	To make up for lower inflow from canals	Technical	Operational	Farmer	Koram, Karaoi
Monitoring of the water system (e.g. reservoirs levels)	To enable a timely response to water stress	Technical	Operational	Farmer, Water User Association	Karaoi
Coordination of water distribution among farmers (water supply rotation)	To enable fair distribution of scarce water resource and of negative impacts	Institutional	Operational	Farmer, Water User Association	Koram, Karaoi
Efficiency improvement (e.g. cleaning or repairing intra-farm canals)	To reduce water losses	Technical	Tactical	Farmer, Water User Association	Koram, Karaoi
Change to less water demanding crops	To reduce water need	Technical	Strategic	Farmer	Karaoi
Adoption of more efficient irrigation technology	To reduce water need	Technical	Strategic	Farmer	Karaoi
Temporary or permanent exit from agriculture (non voluntary)	To avoid risk of further crop failure and loss of livelihood	Economic	Strategic	Farmer	Koram
Establishment of formal responsibilities for water supply infrastructure management	To clarify water supply rules, and enable investment and management	Institutional	Structural	District Council	Koram

^a^(Risbey *et al*. 1999)

While operational adaptation practices in Koram generally corresponded to those in Karaoi, practices with a longer-term (strategic and structural) effect differed, probably reflecting the different challenges and dominant farm profiles. In Karaoi, farmers had tested (or at least considered adopting) more efficient irrigation technology, such as drip irrigation, and also reported shifting cultivation to less water demanding crops, such as alfalfa or safflower. These crops were less profitable, but were generally considered a safe option as they were used to feed livestock on mixed farms, or sold to livestock farms locally. In Koram, which is characterised by smallholding and lower levels of mechanisation, the adoption of more efficient irrigation technologies did not appear to have been tested or considered, possibly because it was beyond the financial capacity of smallholders. The predominance of less economically secure smallholdings in Koram seems to be reflected in non-voluntary exiting from agricultural production, often temporarily, after bankruptcy caused by crop loss as a result of water scarcity and variability of spring and winter frosts. However, despite the relative frequency of bankruptcy and in spite of the extreme droughts of 2010 and 2014, farmers in Koram reported having increased the area under irrigated cultivation in response to demand for food crops.

Koram was characterised by substantial uncertainty over the management responsibilities of supply canals and supply rules, reflected in the application by the regional administration to the national government for formal recognition of authority over so-called ‘unmanaged’ on-farm channels. Once the years-long procedure is completed, and subject to involvement of an investment partner, this may enable investment in canals and associated infrastructure (e.g., water meters), and rule enforcement with structural (decadal) effects.

These adaptation practices (Table 1) do not amount a unified and coherent adaptation strategy. For example, in Koram and among smallholders in Karaoi the voluntary cleaning of on-farm channels seemed an institutionalised practice, while other practices, such as the use of alternative water sources or the adoption of more efficient irrigation systems, were usually reported by medium and large landholders with higher social capital who had access to these sources and technologies.

It was apparent that actors perceived the institutional and political environment and the decaying infrastructural and technical system as fixed boundaries. There was no sign of attempts to challenge those institutional and technical structures, which would constitute a transformative form of adaptation (Feola 2013, 2015) and effectively expand the adaptive space of farmers, WUAs, and local authorities. Most adaptations were therefore operational and appeared to have positive effects on agricultural production in the region. But longer term (structural) adaptation, either social or technical, seemed not to be an option due to the costs of an infrastructural upgrade and the resistance to change of an established top-down decision-making system that allocates responsibilities to lower administrative levels in principle, but actually maintains decision-making (and financial) power at higher levels.

### Entry Points for Adaptation

The workshops revealed two specific entry points for adapting water use in agriculture to climate change. Firstly, the need for infrastructural investment directed towards efficient use of water resources, i.e., prevention of water loss within the system. While financing of infrastructural upgrades and maintenance is a major constraint, actors were hopeful of private investment in this domain. Farmers in Koram gossiped that “the Arabs” might be interested in repairing the MK Kuram. In Karaoi, people had heard that Chinese companies were investing in irrigation for soya production. Limited foreign-backed partnerships were operating in both areas (e.g., seed production, orchard management). This openness to investment is tempered by local worries about government plans to lease land to foreign investors, which has led to popular demonstrations in protest against feared Chinese land-grabs.

Secondly, the need for information on shorter and longer-term weather patterns and for improved communication of such information. Knowledge of climate change forecasting and trends in water balance in the area is poor, and local actors (including farmers and heads of WUAs) are keen to obtain such knowledge. Up-to-date information from the few weather stations in the area is available online; data from river gauges are not freely available; weather and agricultural (but not climate) forecasts go to larger farms who have their own agronomists but not to small producers (Shahgedanova, personal communication). Provision of climate information would enable local actors to better plan, and NGOs to orient their activities towards the sustainability of agriculture in the area. Local organisations are well positioned to communicate to farmers and water users, but there seems to be little coordination among these organisations and higher system levels, as well as lack of trained personnel.

Finally, the attitude of farmers, local administrations, and NGOs (when present) indicates receptiveness to adaptation innovations. Farmers are already working marginal land despite the frequent risk of crop failure. Even with these constraints, farmers seek to adapt and maintain dilapidated infrastructure (e.g., damming sections of a river to create a new water source) and experiment with crop varieties and growing methods (with the support of a local agribusiness centre). Officials within the administration are also active in seeking to bring ‘unmanaged’ canals under management and direct investment for concrete lining of canals (e.g., MK Kuram). Notwithstanding the need to address the more structural constraints, which are often associated with higher administrative levels, the levels of experimentation observed at local level in both villages opens opportunities for adaptation.

## Discussion and Conclusions

Our research was designed to investigate constraints and opportunities for adaptation of agricultural water-use to climate change in southeast Kazakhstan. We found that stakeholders in both study sites were concerned about water supply and poor infrastructure, while concerns with high water prices (Koram) and lack of irrigation technology (Karaoi) reflected the socioeconomic circumstances of farmers of each site. In both sites, actors engaged in a range of adaptation measures and were interested in exploring all available options, including self-organisation, and engaging state and non-state, commercial and non-commercial actors. In particular, water system stakeholders repeatedly raised the issues of financing and improving technical knowledge and capacity. However, despite considerable operational and tactical adaptation there was little structural adaptation.

Constraints are significant and specifically related to the infrastructural and institutional structures. Current irrigation infrastructure is a legacy of the Soviet period and much has deteriorated or been destroyed. Local institutions (WUAs, local authorities, farmers) cannot finance rehabilitation, let alone invest in new infrastructure. Present-day water-management institutions were cobbled together by state actors in response to a liberalising national policy and remain functionally part of the state. We also observed a continued ‘statist’ orientation with workshop participants overwhelmingly identifying constraints as related to government programmes and questions of management. Our findings broadly confirm those of earlier studies in Kazakhstan (Wegerich 2008; Zinzani 2015) and in neighbouring formerly Soviet countries (Hornidge *et al*. 2011; Oberkircher and Hornidge 2011), although the more fragmented farming system and absence of central coordination and agricultural production targets mean that WUAs in South Kazakhstan are more flexible. Nevertheless, the importance of the state to local actors leads to two issues specific to the post-Soviet transformation: the implementation of water management models in practice and the post-Soviet institutional environment.

### Implementation of Water Management Models

An important finding of this research is that aspects of IWRM did not translate well into the Kazakh context. Despite stated intentions to improve water-use efficiency through the introduction of market mechanisms to water management, in the absence of individualised water consumption measurement there is at present no incentive for farmers to economise water consumption. As one farmer put it, “in the season, we use as much as they [the BAK] give, we can’t get enough.” Although assessed volumetrically and priced at market rates, water is still apportioned on the basis of Soviet-style consumption norms (calculated on the basis of irrigation method, crop type and area under cultivation, and including acceptable water losses of up to 33%; actual water losses may be significantly higher). Without effective water metering, the present system merely perpetuates inefficient water use (at increased costs): farmers seem to have accepted significant price hikes in 2010 without adjusting consumption behaviour. Lack of accurate metrification may explain why farmers prioritised the issues of ‘water supply’ and ‘water prices’ over ‘water losses’ since losses are unmeasured (cf. Biswas 2004; Solokov 2006; Meinzen-Dick 2007; Mollinga *et al*. 2007).

IWRM was implemented with the intention that markets would make up for state divestment from irrigation (Burger 1998). Our results confirm earlier studies in Kazakhstan that found, although effective in coordinating water use and on-farm canal maintenance, in market conditions of full-cost recovery WUAs struggled to meet costs and maintain already deteriorated infrastructure and were incapable of financing further investments (Burger 1998; Johnson 1998; Rosen and Strickland 1998; Wegerich 2008; Zinzani 2015). WUAs are unable to raise sufficient funds from farmers and subventions to cover operating costs and basic maintenance without relying on farmers’ voluntary labour. Farmers’ margins are too small and rural land markets insufficiently developed to provide collateral for loans to finance infrastructural investments (business loans can only be secured with property in Almaty^5^). The role of WUAs thus remains limited to managing annual water flow, coordinating the share of irrigation water among farmers and maintaining on-farm infrastructure. For small-scale farming, at least, it would be unreasonable to expect a thriving market for water to develop, certainly not one that would permit the infrastructural upgrading of the system that is necessary for improved productivity and adaptation to water stress. This could change with big investments in large-scale farms (as is occurring with Chinese investment in soya in nearby Sorbulaq).

We found that farmers and local state officials are willing to engage at a range of levels to overcome challenges in water provision, agricultural production, and sales; as one farmer commented: “Everyone must do things for themselves. If you work, things will turn out well, but we have to do it for ourselves.” However, the reality is that farmers in Koram and Karaoi have limited capacity. The state and its local and semi-privatised organs (*akimat*, BAK, PK, WUA) is the main institutional actor and farmers’ attitudes reflect this. Indeed, the continued statist orientation of farmers with respect to infrastructure provisioning may not be so much a reflection of Soviet culture or nostalgic attitudes as economic pragmatism.

Infrastructural investment directed towards efficient use of water resources would probably also lead to intensification of demand (‘rebound effect’ Alcott (2005)) and would not necessarily make the system more resilient to extreme weather events such as droughts. It would also not reduce the irrigation system’s continued vulnerability to weak administration, untimely completion of contracts, and erratic delivery of water. But it is an essential component of future agricultural planning, which might include re-designation of irrigated and other agricultural zones to reflect future climate and water availability projections rather than the Soviet-era benchmarks that currently inform planning (Hornidge *et al*. 2013).

### Adaptation to Climate Change in Post-Soviet Institutional Settings

Kazakhstan’s limited climate change policy framework remains little implemented beyond concept documents (Lioubimtseva and Henebry 2009; Yesserkepova 2010). One factor in this may be the specific post-Soviet bureaucratic environment within which policy is formulated and enacted.

Sociological research has shed light on the considerable repertoire of informal practices that characterise post-Soviet bureaucracies, and which animate an otherwise brittle set of hierarchical and centralised institutions (Willerton 1992; Sehring 2009; Ledeneva 2006; Kononenko and Moshes 2011). Our research identified several instances where informal practices compensated for deficiencies in formal water management (e.g., welding sluices shut, voluntary maintenance, damming a river) with tacit acceptance, if not support, from local officials.

Yet these workarounds solve only specific problems and do not contribute to the resolution of broader systemic adaptation issues. Structural adaptation measures may be impeded by a closed, top-down bureaucracy that delegates little responsibility or funds to local-level administration. In consequence, an often internationally funded NGO sector seems to be attempting to fill a significant capacity gap in state administration. Although these organisations may be effective in supporting local authorities and populations anecdotal evidence suggests that this may have resulted in a parallel system that achieves substantive outcomes while perpetuating the inertia and incapacity of state bureaucracy.

The range of local innovations we encountered in this study nevertheless testifies to farming system actors’ concern for and engagement with water issues within tight constraints. We find that efforts to support and promote *structural* adaptation measures cannot take a normative or ‘out of the box’ approach, but must work with and within constraints (including institutional and infrastructural path-dependence) inherent in post-Soviet governance systems, on which basis strategic interventions allow for necessary and locally appropriate institutional adaptation. Future place-based studies of water management can make an important contribution to better understanding the challenges of climate change adaptation in such institutional contexts.

## Electronic Supplementary Material

ESM 1 (PDF 189 kb)

## Footnotes

1 Hornidge *et al*. (2011) encountered similar issues in Uzbekistan, including endemic corruption in the public sector, limited sense of ownership regarding water user associations, and little involvement in water management.

2 Some studies show how farmers and other actors navigate the above-mentioned dysfunctions in other ex-Soviet countries (see e.g., Hornidge *et al*. 2013 and Oberkircher and Hornidge 2011). Importantly these authors did not find evidence of efficiency being a value for farmers, as Western economic rationales with regard to scarce resources are not part of the local concept of water management. Discursive practices mediate this discrepancy, thus maintaining the system by verbally affirming formal arrangements in the face of informal practices. Informal practices (e.g., installing illicit water pumps) may facilitate water management in the face of structural deficiencies, but they also reinforce a divide between official and actual practice, which hinders learning and constitutes a barrier to institutional adaptation.

3 Field research focused on the water system around Karaoi village, but inevitably also took into account farmers’ landholdings in the wider district.

4 The Soviet term *vnutrikhozyaystvennyi kanal* [intra-farm channel] refers to the network of irrigation channels that were internal to the state farm. Following privatisation these channels supply fields of different farmers and would be more appropriately described as inter-farm channels. We use the term “on-farm channel” to indicate that farmers are considered to have responsibility for them.

5 In both field sites, it is possible to identify the emergence of commercial agriculture with investments from the city. This was more pronounced in Karaoi, but was occurring on a small scale with regards to orchard production in Koram.
