# Supplementary material for: Adapting Agricultural Water Use to Climate Change in a Post-Soviet Context: Challenges and Opportunities in Southeast Kazakhstan
Source: Hum Ecol Interdiscip J. 2017 Oct 30;45(6):747–62. doi: 10.1007/s10745-017-9947-9 (PMC5698361; doi:10.1007/s10745-017-9947-9)
Supplement: Supplementary file 1 — (PDF 189 kb) [file 10745_2017_9947_MOESM1_ESM.pdf]

HUMAN ECOLOGY

Electronic supplementary material

Adapting agricultural water use to climate change in a post-Soviet context: challenges and opportunities in Southeast Kazakhstan

DOI: 10.1007/s10745-017-9947-9

Authors: Tristam Barrett [1 2], Giuseppe Feola [1 *], Marina Khusnitdinova [3], Krylova Viktoria [3]

[1] University of Reading, Department of Geography and Environmental Science

[2] Max Planck Institute for Social Anthropology

[3] Kazakh Institute of Geography

* Corresponding author

Department of Geography and Environmental Science, University of Reading, Whiteknights, RG6 6AB Reading, United Kingdom, g.feola@reading.ac.uk, 0044 118 3787496

Table A1. Challenges to water use in agriculture in Koram and Karaoi.

Study site	Challenge	Problem area	Mentions in individual cards (Exercise 1)	Total mentions in group cards (Exercise 2)	Small holders: Mentions	Small holders: Ranking positions	Middle and large holders: Mentions	Middle and large holders: Ranking positions	NGOs and agrobusinesses: Mentions	NGOs and agrobusinesses: Ranking positions	Scientists: Mentions	Scientists: Ranking positions	State structures: Mentions	State structures: Ranking positions
Koram	Inadequate water supply	Water supply	9	2	-	-	1	4	1	3	-	-	-	-
	Untimely water supply		11	4	2	1, 5	2	1, 5	-	-	-	-	-	-
	Water supply rules		11	3	1	3	-	-	-	-	-	-	2	2, 4
	Poor infrastructure/maintenance	Poor infrastructure	20	3	-	-	1	2	-	-	1	2	1	1
	No/bad consumption measurement		8	3	1	2	-	-	1	5	-	-	1	3
	High water prices	High water prices	4	3	1	4	1	3	1	2	-	-	-	-
	Bad soil cultivation	Other	1	1	-	-	-	-	-	-	1	5	-	-
	Climate change		2	1	-	-	-	-	-	-	-	-	1	5
	Irrigation practices		3	-	-	-	-	-	-	-	-	-	-	-
	Lack of knowledge		6	-	-	-	-	-	-	-	-	-	-	-
	Lack of irrigation technology		2	-	-	-	-	-	-	-	-	-	-	-
	Land use		4	-	-	-	-	-	-	-	-	-	-	-
	Little penetration of new crops		1	1	-	-	-	-	-	-	1	4	-	-
	No problem		2	-	-	-	-	-	-	-	-	-	-	-
	No science-user connection		1	1	-	-	-	-	-	-	1	3	-	-
	Other		5	-	-	-	-	-	-	-	-	-	-	-
	Reducing water stock		2	1	-	-	-	-	-	-	1	1	-	-
	Salinisation		1	-	-	-	-	-	-	-	-	-	-	-
	Water losses		5	2	-	-	-	-	2	1, 4	-	-	-	-
	Water quality		2	-	-	-	-	-	-	-	-	-	-	-

Study site	Challenge	Problem area	Mentions in individual cards (Exercise 1)	Total mentions in group cards (Exercise 2)	Small holders: Mentions	Small holders: Ranking positions	Middle and large: Mentions	Middle and large: Ranking positions	NGOs: Mentions	NGOs: Ranking positions	Agrobusinesses: Mentions	Agrobusinesses: Ranking positions	State structures: Mentions	State structures: Ranking positions
Karaoi	Inadequate water supply	Water supply	16	4	-	-	3	1, 2, 3	-	-	1	3	-	-
	Poor infrastructure/maintenance	Poor infrastructure	21	5	1	1	-	-	1	2	1	2	2	1, 3
	Lack of irrigation technology	Lack of technology	7	3	1	5	-	-	-	-	-	-	2	4, 5
	Climate change	Other	2	1	-	-	-	-	-	-	1	4	-	-
	Corruption		1	-	-	-	-	-	-	-	-	-	-	-
	High water prices		3	1	-	-	-	-	-	-	1	1	-	-
	Irrigation practices		3	-	-	-	-	-	-	-	-	-	-	-
	Labour intensity		1	-	-	-	-	-	-	-	-	-	-	-
	Lack of financial resources		1	-	-	-	-	-	-	-	-	-	-	-
	Lack of knowledge		1	-	-	-	-	-	-	-	-	-	-	-
	Lack of technical personnel		2	1	-	-	-	-	1	5	1	5	-	-
	Land use		2	-	-	-	-	-	-	-	-	-	-	-
	Loss of Soviet irrigation system		1	1	-	-	1	5	-	-	-	-	-	-
	No/bad consumption measurement		2	1	-	-	-	-	1	4	-	-	-	-
	Non rational water use		1	1	-	-	-	-	1	1	-	-	-	-
	Other		3	-	-	-	-	-	-	-	-	-	-	-
	Water supply rules		11	2	1	2	-	-	1	3	-	-	-	-
	Untimely water supply		1	1	1	4	-	-	-	-	-	-	-	-
	Water losses		2	2	1	3	-	-	-	-	-	-	1	2
	Water quality		3	1	-	-	1	4	-	-	-	-	-	-
